# Exploring the G-quadruplex binding and unwinding activity of the bacterial FeS helicase DinG

**DOI:** 10.1038/s41598-023-39675-5

**Published:** 2023-08-03

**Authors:** Elisa De Piante, Federica D’Aria, Luisa M. R. Napolitano, Jussara Amato, Simone Pirrello, Silvia Onesti, Concetta Giancola

**Affiliations:** 1 Structural Biology Laboratory, Elettra-Sincrotrone Trieste S.C.p.A, 34149 Trieste, Italy; 2 Department of Pharmacy, University of Naples Federico II, 80131 Naples, Italy

**Keywords:** Biophysics, Chemistry

## Abstract

Despite numerous reports on the interactions of G-quadruplexes (G4s) with helicases, systematic analysis addressing the selectivity and specificity of each helicase towards a variety of G4 topologies are scarce. Among the helicases able to unwind G4s are those containing an iron-sulphur (FeS) cluster, including both the bacterial DinG (found in *E. coli* and several pathogenic bacteria) and the medically important eukaryotic homologues (XPD, FancJ, DDX11 and RTEL1). We carried out a detailed study of the interactions between the *E. coli* DinG and a variety of G4s, by employing physicochemical and biochemical methodologies. A series of G4-rich sequences from different genomic locations (promoter and telomeric regions), able to form unimolecular G4 structures with diverse topologies, were analyzed (c-*KIT1*, *KRAS*, c-*MYC*, *BCL2*, Tel_23_, *T30695*, *Zic*1). DinG binds to most of the investigated G4s with little discrimination, while it exhibits a clear degree of unwinding specificity towards different G4 topologies. Whereas previous reports suggested that DinG was active only on bimolecular G4s, here we show that it is also able to bind to and resolve the more physiologically relevant unimolecular G4s. In addition, when the G4 structures were stabilized by ligands (Pyridostatin, PhenDC3, BRACO-19 or Netropsin), the DinG unwinding activity decreased and in most cases was abolished, with a pattern that is not simply explained by a change in binding affinity. Overall, these results have important implications for the biochemistry of helicases, strongly suggesting that when analysing the G4 unwinding property of an enzyme, it is necessary to investigate a variety of G4 substrates.

## Introduction

Although DNA G-quadruplex (G4) structures are often defined as “non-canonical”, they are ubiquitous in the genomes of various organisms^1^. G4s are formed by guanine-rich sequences that folds into guanine tetrads stabilized by Hoogsteen hydrogen bonds and monovalent cations. They have been extensively studied by NMR^2,3^, X-ray crystallography^4,5^ and other physicochemical methodologies^6,7^, in terms of structural topologies and dynamics. G4s exhibit considerable thermodynamic stability and act as “rope knots” that challenge and modulate processes like DNA replication, DNA repair and transcription, as well as acting as recruiting hubs for transcription factors and other protein factors^8^. Although the physiological roles of G4s are not fully understood, a growing body of evidence suggests that G4 formation and regulated unwinding are critical processes within bacterial and eukaryotic cells, and in the latter their dysregulation has a major impact in genome stability and cancer onset. G4s can therefore be exploited as targets for selective gene suppression/modulation and there is an ever-growing plethora of identified and characterized G4-binding and G4-stabilizing small molecules^9,10^, usually containing hydrophobic, aromatic, planar moieties^11–16^. Whereas most efforts have so far focused on eukaryotic G4s, there is increasing evidence that G4s are also important in viruses and bacteria and are therefore attractive targets for the development of antiviral and antibiotic drugs^17,18^.

Numerous helicases have been shown to bind and unravel G4 structures, and hence play a key role in G4 metabolism. Among the helicases able to unwind G4s, an important part is played by the iron-sulphur (FeS) helicase family, which is conserved from bacteria to humans. In archaea and eukaryotes, FeS helicases are ubiquitous, with human cells including four paralogues (XPD, FancJ, DDX11 and RTEL1), which have important roles in DNA replication, DNA repair, telomere metabolism, and sister chromatid cohesion, and are involved in genetic diseases and cancer metabolism^19^.

In bacteria the situation is more confused: most, but not all, bacteria contain at least one protein sharing similarity to the archaeal and eukaryotic FeS helicases, but in a subset of bacteria the helicase is fused to an N-terminal exonuclease-like domain, and in some cases (as in *Staphylococcus aureus*) the helicase domain has lost the FeS cluster and thus the ability to unwind DNA^20^. The prototype of a bacterial functional FeS helicase is DinG from *Escherichia coli*. It acts as a 5′-3′ DNA helicase^21^ and unwinds not only DNA-forked structures, but also DNA-RNA hybrid duplexes, flap substrates, d-loops and R-loops^22^. The crystal structure of DinG in complex with ssDNA (in the presence and absence of an ATP analogue) shows the fold of the molecule and suggests a mechanism for 5′ to 3′ translocation^23^. Although deletion of the corresponding gene confers very mild sensitivity to UV and DNA damaging agents, a potential physiological role of DinG in the dissolution of R-loops arising from the collision of the replication and transcription machineries has been shown^24^.

In addition to unwinding DNA/RNA hybrids and R-loops, both *E. coli* and *M. tuberculosis* DinG were shown to be able to resolve G4 DNA structures. In particular, *E. coli* DinG was tested with both unimolecular and bimolecular G4s, and was shown to be able to unwind only the latter^25^; for *M. tuberculosis* DinG only bimolecular and tetramolecular G4s were tested^25,26^.

The ability to bind and unwind G4s is shared across most members of the FeS helicase family, including FANCJ, RTEL1 and DDX11^27–31^; this makes DinG an attractive model system for the function of the more challenging and medically relevant eukaryotic homologues, besides being itself a useful target, due to the presence in a number of widespread bacterial pathogens.

To harness the potential of G4s as drug targets is essential to understand the interplay between G4 and helicases, and the impact of G4 binders on the G4/helicase interaction. The understanding of the molecular determinants for helicase:G4 interactions have been strongly hindered by a lack of structural data on the macromolecular complexes. Until very recently the only three-dimensional experimental model for a G4 bound to a helicase was that of DHX36, a member of the DExD/H box helicase family with an important role in G4 metabolism^32^. A more recent structure of the Pif1 helicase bound to a G4 shows an unexpected arrangement, with the G4 wedged between two Pif1 monomers, with very few specific interactions between the protein and the core of the G4^33^.

Due to the paucity of structural data, most of the information is based on biophysical and physicochemical analysis. However, most studies tend to focus on one or two G4s, whereas systematic studies on the interaction modes and energetics of helicases, G4 and G4 ligands are rare^34–38^. Here, we study the binding and unwinding activities of the FeS helicase DinG from *E. coli* towards a number of unimolecular G4s, and the impact of four G4 ligands, namely Pyridostatin (PDS), PhenDC3, BRACO-19 and Netropsin. Physicochemical and biochemical methodologies were employed to analyse the energetics of molecular interactions and dynamical behaviour of G4/DinG complexes with and without G4 binders. Surface Plasmon Resonance (SPR) provided information on the thermodynamic and kinetic aspects of (bio)molecular interactions: reaction rates and equilibrium constants of G4/helicase interactions were determined, and the effects of the G4 ligands were followed in real time. In parallel, a fluorescence-based helicase assay allowed to monitor the unwinding of DinG towards a variety of G4 structures and G4/ligand complexes.

Our results provide a systematic overview of the interactions between a FeS helicase and a variety of G-quadruplexes with different topologies and different cellular roles, and further examine the effect of G4 ligands on these interactions. Contrary to previous findings, we show that DinG is able to recognize physiologically relevant unimolecular G4s characterized by different folding topologies, and to unwind them with a degree of specificity. We find that there is not an exact correlation between binding and unwinding, in line with the experimental structural data for DHX36 that show that the recognition of G4 involves a region that is distinct from the unwinding active site^32^.

## Materials and methods

### Cloning

The full-length gene encoding *E. coli* DinG was amplified from *E. coli* genomic DNA and cloned into a pNIC28-Bsa4 vector using the LIC method to express the target protein with a cleavable N-terminal Hexa-histidine tag. The pNIC28-Bsa4 vector was a gift from Opher Gileadi (Addgene plasmid #26103; http://n2t.net/addgene:26103; RRID:Addgene_26103)^39^.

### Protein expression and purification

The DinG construct was expressed into *E. coli* B834 (DE3) competent cells. Cells were grown at 37 °C in Luria Bertani (LB) broth medium supplemented with 50 μg/ml of kanamycin and 10 μM FeCl_3_, to an optical density (OD_600_) of 0.8. Protein expression was induced by the addition of 0.15 mM isopropyl 1-thio-β-d-galactopyranoside (IPTG) and incubated overnight at 18 °C at 150 rpm. The purification protocol is very similar to that reported by Chen et al.^32^. Briefly, after harvesting, cells were resuspended in Lysis buffer (20 mM Tris–HCl pH 8.0, 5 mM imidazole, 1 M NaCl), lysed by sonication and centrifuged at 4 °C for 60 min at 18,000*g.* The supernatant was added to 1.2 mL Ni–NTA resin (Qiagen) pre-equilibrated with Lysis Buffer and the His-tagged DinG eluted with 300 mM imidazole. The eluate was collected, and the salt concentration was reduced to 150 mM NaCl. The solution was applied to a 1 mL Heparin FF column (Cytiva), pre-equilibrated with Heparin buffer (20 mM Tris pH 8.0, 20% Glycerol, 0.5 mM TCEP). The column was washed with 10 CV of Heparin buffer and the protein was eluted with a linear gradient over 40 CV to a concentration of 700 mM NaCl. Appropriate fractions were pooled and concentrated. As a final polishing step, the protein was applied to a Superdex 200 10/300 GL column (GE Healthcare) with a Gel Filtration Buffer (20 mM Tris pH 8.0, 300 mM NaCl, 0.5 mM TCEP and 10% Glycerol). Protein was flash frozen in liquid nitrogen and stored at − 80 °C.

### Oligonucleotides and ligands

All oligonucleotides used in this study were purchased as HPLC-purified sequences from Biomers.net GmbH (Ulm, Germany). Table 1 reports the sequences of the eukaryotic G4 used for the binding assays, while Supplementary Table S1 shows the sequence of the bacterial G4 tested as a control. Supplementary Table S2 lists the DNA substrates and capture strands used in the G4 helicase assays^35^. Supplementary Table S3 shows the substrates used for the unwinding assay with a DNA fork, used as a control.

**Table 1:** List of the human G4-forming sequences utilized in the study.

DNAs	Sequence	Topology, PDB-ID	Source
c-*KIT*1	5′-A**GGG**A**GGG**C**G**CT**GGG**AGGAG**GG**-3′	Parallel, 2O3M	Promoter
c-*MYC*	5′-A**GGG**T**GGG**TA**GGG**T**GGG**T-3′	Parallel, 1XAV	Promoter
*KRAS*	5′-A**GGG**C**GG**T**G**T**GGG**AATA**GGG**AA-3'	Parallel, 5I2V	Promoter
*T30695*	5′-**GGG**T**GGG**T**GGG**T**GGG**T-3′	Dimeric Parallel, 2LE6	Anti-HIV integrase aptamer
*BCL*2	5′-**GGG**CGC**GGG**AGGAATT**GGG**C**GGG**-3′	Hybrid [3 + 1], 2F8U	Promoter
Tel_23_	5′-TA**GGG**TTA**GGG**TTA**GGG**TTA**GGG**-3′	Hybrid [3 + 1], 2HY9	Telomere
*Zic*1	5′ GGT**GGGGGGGCGGGGG**AGGCC**GGG**-3′	Parallel	non-template strand of transcribed gene

Bold G indicate the guanine base that are part of the quadruplex structure.

The G4 ligands PDS [4-(2-aminoethoxy)-N2,N6-bis(4-(2-aminoethoxy)quinolin-2-yl)pyridine-2,6-dicarboxamide], PhenDC3 [3,3′-[1,10-Phenanthroline-2,9-diylbis(carbonylimino)]bis[1-methylquinolinium] 1,1,1-trifluoromethanesulfonate, BRACO-19 (N,N’-(9-[(4-(dimethylamino)phenyl)amino]acridine-3,6-diyl)bis(3-(pyrrolidin1-yl)propanamide)) and Netropsin (N-[5-[(3-amino-3-iminopropyl)carbamoyl]-1-methylpyrrol-3-yl]-4-[[2-(diaminomethylideneamino)acetyl]amino]-1-methylpyrrole-2-carboxamide) were purchased from Sigma Aldrich (Merck KGaA, Darmstadt, Germany), along with all chemicals, reagents and solvents used for this study.

### DNA sample preparation

The oligonucleotides used for SPR experiments (Table 1 and Supplementary Table S1) were prepared by dissolving the lyophilized DNAs in potassium phosphate buffer (10 mM KH_2_PO_4_/K_2_HPO_4_, 50 mM KCl, at pH = 7.5). The concentration of each oligonucleotide was evaluated by UV measurement at 90 °C, using molar extinction coefficient values at 260 nm. The solutions were heated at 95 °C for 5 min and then slowly cooled to room temperature and stored at 4 °C overnight before use.

For the two-step fluorescence-based helicase assay, the DNA systems were annealed at 1 μM concentration by preparing a mixture of 1 μM Dabcyl-labelled oligonucleotide and 0.85 μM FAM-labelled oligonucleotide in 20 mM Tris–HCl buffer (pH 7.2, 5 mM MgCl_2_, 1 mM KCl and 99 mM NaCl). The 1.2-fold excess of Dabcyl-labelled strand was used to assure the total hybridisation of the FAM-labelled strand and therefore to achieve the maximum quenching of the fluorescent signal (Supplementary Table S2). Solutions were denatured at 95 °C for 5 min and then slowly cooled to room temperature and stored at 4 °C overnight before use. For the FRET helicase assay, a fork DNA (Supplementary Table S3) was prepared using the following protocol: the fluorescent strand (D1) and the complementary strand (D2) were annealed at a 1:2.7 M ratio in 10 mM Tris–HCl buffer (pH 7.5, 50 mM KCl) by heating at 95 °C for 5 min followed by slow cooling to room temperature.

### Circular dichroism experiments

Circular dichroism (CD) experiments were performed on a Jasco J-815 spectropolarimeter equipped with a PTC-423S/15 Peltier temperature controller. CD spectra were recorded at 20 °C in the wavelength range of 230–340 nm and averaged over three scans. The following parameters were used: 100 nm min^−1^ scan rate, 0.5 s response time and 1 nm bandwidth. A 2 μM oligonucleotide concentration was used. CD melting experiments were carried out in the 20–90 °C temperature range at 1 °C min^−1^ heating rate, following changes of CD signal at the wavelength of maximum intensity of c-*KIT*1 (264 nm). CD melting experiments were recorded both in the absence and presence of each ligand. DNA/ligand mixtures were obtained by adding 1 or 2 mol equiv. (2 or 4 μM) of PDS, PhenDC3, BRACO-19 and Netropsin to the folded G4 structure.

### SPR analysis

SPR experiments were carried out on a Biacore X100 (GE Healthcare, Uppsala, Sweden). DinG helicase was immobilized on a research-grade CM5 sensor chip using the amine-coupling chemistry and the HBS-EP as running buffer (10 mM HEPES, 150 mM NaCl, 3 mM EDTA, 0.005% surfactant P20, pH 7.4) as described elsewhere^40–42^. The protein (100 μg/mL in 10 mM sodium acetate, pH 4.5) was immobilized on the sample flow cell, leaving the reference cell as blank. Single-Cycle Kinetics (SCK) experiments were performed at 25 °C by injecting increasing concentrations of DNA samples (from 0.062 to 1 μM) at a flow rate of 30 μL/min. SCK experiments were also performed on DinG-immobilized sensor chip flowing the c-*KIT*1 G4 in the presence of each of the four selected G4 ligands (BRACO-19, PhenDC3, Pyridostatin, and Netropsin). Increasing concentrations of the [G4:ligand] complexes (1:1 molar ratio) were flowed on the immobilized protein (from 0.062 to 1 μM) at a flow rate of 30 μL/min. For these experiments, a running buffer consisting of 10 mM KH_2_PO_4_/K_2_HPO_4_ solution (pH 7.5) containing 50 mM KCl was used, and association and dissociation times were set at 120 and 600 s, respectively. Curves obtained on the reference surface were subtracted from those recorded on the protein-functionalized surface, to eliminate the effects of refractive index changes due to the buffer. Data were fitted to a 1:1 kinetic interaction model, using the global data analysis option available within the BiaEvaluation software (GE Healthcare, Uppsala, Sweden) provided with the instrument.

### G4 helicase assay

G4 helicase assay was performed following the procedure described by Mendoza et al.^35^. Helicase reactions were carried out in triplicate in 384-well plates (Corning, 384-well, black, low volume, flat bottom, non-binding surface) at 25 °C and fluorescence monitored by a microplate reader (Tecan Infinite F200 PRO). Every replicate contained a 30 μl solution containing 40 nM FAM-dabcyl system (S-c-*MYC*, S-c-*KIT*1, S-*BCL*2, S-*KRAS*, S-Tel_23_, S-*T30695* or S-*Zic1*) previously annealed, 20 nM of *E. coli* DinG and 200 nM of Trap oligonucleotide (unlabelled, complementary to the FAM-labelled strand). Next, 5 mM of ATP solution was added to every well and the fluorescence emission was recorded every 30 s (the excitation wavelength was set at 485 nm and the emission wavelength at 535 nm). Once the maximum emission was reached and the signal was stable (30 min), 2 μM of a strand complementary to the dabcyl-labelled sequence (C–c-*MYC*, C–c-*KIT1*, C-*BCL*2, C-*KRAS*, C-Tel_23_, C- *T30695* or C-*Zic1*) was added to every well and emission was monitored every 30 s for 30 min.

### G4 helicase assay in the presence of G4-ligands

The same procedure as used for G4 substrate screening was applied for the G4-ligand screening. A 30-μl solution of 40 nM G4 system, 20 nM *E. coli* DinG, 200 nM Trap single-strand and 1 μM of selected ligand was prepared in 20 mM Tris–HCl buffer (pH 7.2, 10 mM MgCl_2_, 1 mM KCl and 99 mM NaCl). ATP and the single-stranded complementary sequence were added following the same procedure as above. The emission was monitored every 30 s.

### Fork DNA helicase assay

The helicase activity with a forked DNA was measured by using fluorescence resonance energy transfer (FRET), by using a substrate with a fluorophore (6-FAM) on one strand and a quencher (BHQ1) on the other (Supplementary Table S3). The assay was performed in 50 mM Tris–HCl pH 7.5, 1 mM DTT, 0.1 mg/mL BSA, 5 mM MgCl_2_ and 50 mM KCl, with 10 nM substrate, 5 mM ATP and 120 nM capture strand (Cap1, Supplementary Table S3) in 30 μl of reaction volume. The unwinding reaction was started by incubating 20 nM of the purified protein in the reaction mixture. The reaction mixture was incubated at 25 °C for 30 min. The fluorescence intensity was recorded using Infinite F200 PRO TECAN instrument. To have a measure of 100% unwinding the reaction was incubated at 95 °C and measured. The assay was done in triplicate. The percentage of unwinding was calculated and plotted using GraphPad-Prism software.

## Results and discussion

The DinG helicase was cloned and expressed in *E. coli* cells, supplemented with FeCl_3_. The protein was purified to homogeneity by Ni-affinity, followed by heparin and size exclusion chromatography: concentrated protein solutions maintained a yellow colour throughout the whole purification protocol, consistent with the retention of the FeS cluster, necessary for the activity. This is in line with the observed stability of the FeS cluster, that was shown to be oxygen-resistant^43^.

A systematic study of the interaction between recombinant DinG and a variety of G4s was then carried out. A panel of G4s was chosen to explore different G4 topologies (parallel/hybrid), with different loop lengths, and different biological relevance (i.e. G4 found in promoters or at telomeres). Although previous studies on *E. coli* DinG showed that the protein unwinds bimolecular but not unimolecular G4s^25^, we choose to focus on unimolecular G4s for two reasons: (1) although some evidence for a role of bimolecular species has recently emerged^44^, the general consensus is that these are likely to be the exception rather than the rule, as a large number of bimolecular G4s (such as the number of G4 that are detected in vivo) would raise a serious topological problem in the cellular context; (2) detecting the unwinding of unimolecular G4s by gel-based assay can be tricky, due to the difficulties in separating the folded/unfolded species, biasing the results towards the easier-to-handle bimolecular or tetramolecular quadruplexes.

The G4-forming sequences chosen for the present study are reported in Table 1 and in Supplementary Table S1. They comprise the 23-mer truncation of the human telomeric G4-forming sequence (Tel_23_), a sequence from the non-template strand of the transcribed regions of the *Zic*1 gene, and the G4-forming sequences from the oncogene promoters of *KRAS*, c-*MYC*, c-*KIT1* and *BCL*2. In particular, *BCL2* and Tel_23_ sequences formed hybrid [3 + 1] G4 structures containing three parallel and one antiparallel G-strands, whereas the G4s from *KRAS*, c-*MYC, c-KIT*1 and *Zic*1 form all-parallel G4s^25,45–48^. In addition, a G-rich sequence forming a dimeric all-parallel-stranded G-quadruplex structure stacked via the 5′-to-5′ interface, named *T30695* (or *T30923*)^49^, and three G-rich sequences from the bacterial genome of *E. coli* all forming unimolecular parallel G4 structures, were included in the study^50^. The proper folding of all these DNA sequences into G4 was verified by CD analysis (Supplementary Figs. S1, S2). Finally, two different control sequences have been considered, the *DNA-fork,* selected as canonical helicase substrate, and the *G-scramble*, a G-rich oligonucleotide not able to assemble into a G4 structure (Supplementary Table S3).

**Figure 1:**
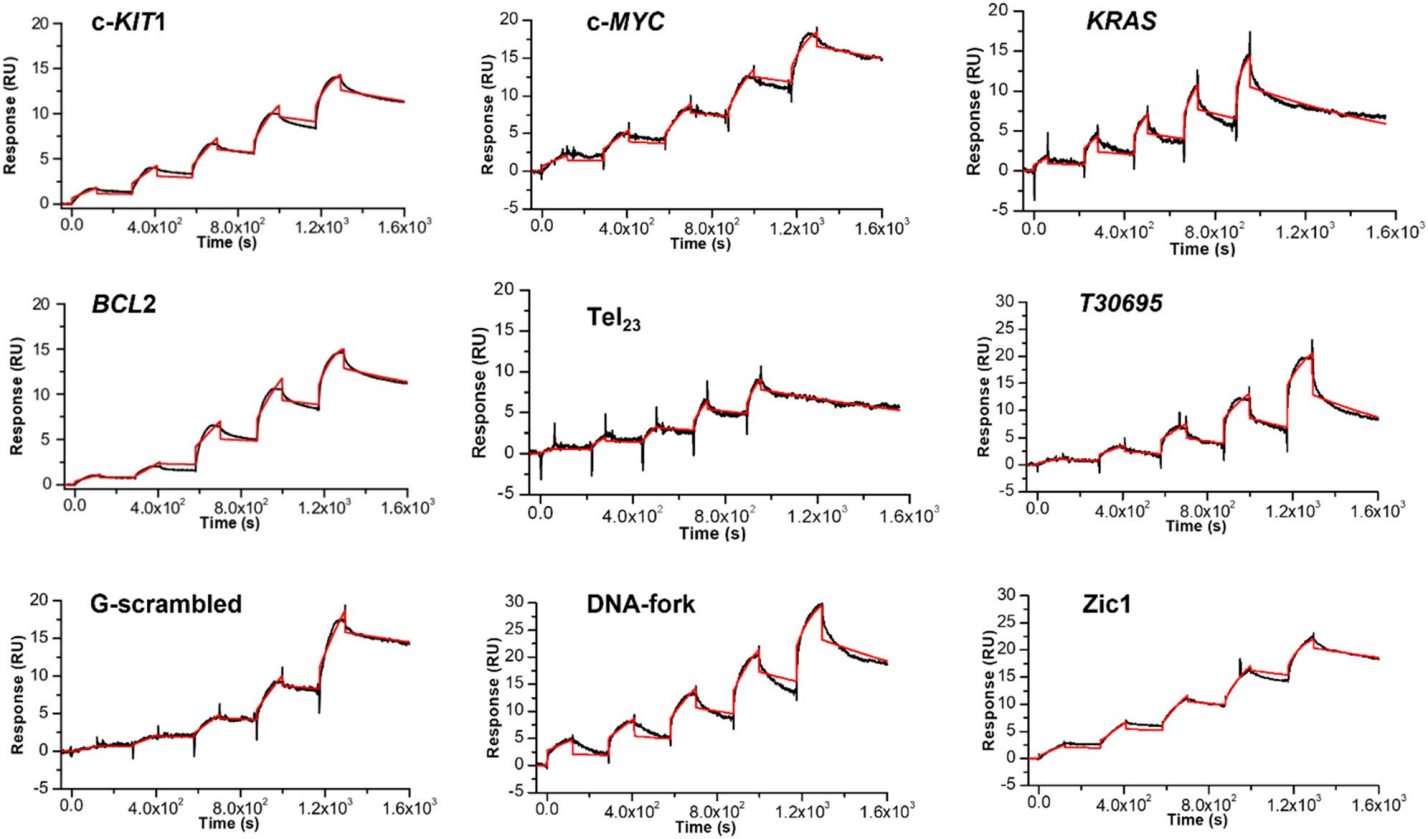
Time evolution SPR sensorgrams obtained at 25 °C by injection of increasing concentrations of each investigated DNA (from 0.062 to 1 µM) on the chip-immobilized DinG helicase.

A physicochemical analysis of the interaction of the investigated DNAs with the DinG helicase was carried out by SPR experiments. Compared to other techniques to study DNA–protein interactions, SPR provides valuable information about the affinity of interactions, with important insights into the kinetic parameters, by measuring the rate constants of association and dissociation (*k*_on_ and *k*_off_,), from which the equilibrium dissociation constant (*K*_D_) can be estimated. Here, SCK method was used, as it allows to perform the regeneration step (which is critical for proteins) only at the end of the complete binding cycle, thus better preserving the integrity of the immobilized protein^40^. Figure 1 shows SPR sensorgrams obtained by injecting increasing concentrations of DNAs (analyte) over the DinG-immobilized sensor-chip by using the SCK. A response proportional to the concentration of injected analyte solution was observed in almost all sensorgrams (Fig. 1), thus indicating the specificity of DNA/protein interactions. Sensorgrams were fitted to 1:1 model to obtain the kinetic rate constants, *k*_on_ and *k*_off_, while the equilibrium dissociation constants (*K*_D_) were calculated as a ratio between *k*_off_ and *k*_on_ (Table 2).

**Table 2:** Kinetics and thermodynamic parameters for the interaction of DinG helicase with different DNAs*.*

	*k*_on_ (M^*–*1^ s^*–*1^) ^a^	*k*_off_ (s^*–*1^)^a^	*K*_D_ (nM)^b^
c-*KIT*1	1.2 × 10^4^	3.3 × 10^–4^	28.9
c-*MYC*	1.1 × 10^4^	3.4 ×10^–4^	30.6
*KRAS*	2.1 × 10^4^	9.6 ×10^–4^	46.4
*BCL2*	2.4 × 10^4^	5.0 × 10^–4^	21.0
Tel_23_	1.5 × 10^4^	1.5 × 10^–4^	9.4
*Zic1*	1.3 × 10^4^	3.0 × 10^–4^	22.8
*T30695*	7.8 × 10^3^	1.3 × 10^–3^	163.4
*G-scramble*	2.3 × 10^3^	2.9 × 10^–4^	126.1
*DNA-fork*	1.1 × 10^4^	6.1 × 10^–4^	55.9

^a^Errors were within 5%.

^b^Errors were within 10%.

The results of the SPR analysis indicate that DinG binds to the investigated G4s with an affinity similar or better to that observed for the *DNA-fork* substrate. The highest affinity value is observed for the interaction with the Tel_23_, which forms a hybrid G4 structure as major conformation, followed by the *BCL2*, which folds in a hybrid G4 structure as well. Lowest affinity values are observed in the case of DinG interaction with the *T30695* sequence, which forms a dimeric all-parallel stranded G4 structure with single-nucleotide containing loops, as well as with the *G-scramble* sequence, which does not form any G4 or other secondary structures, suggesting that DNA recognition by the protein is dependent on the peculiar G4 conformation.

Concerning the kinetic parameters, the values of both *k*_on_ and *k*_off_ differ the most in the case of *T30695* and *G-scramble*, resulting in lower binding affinity of these DNAs to the protein.

Furthermore, since DinG is an *E. coli* enzyme, three different G4-forming sequences from the DNA of *E. coli* were also considered for the interaction with this helicase^50^. SPR sensorgrams were recorded under the same conditions used for the other G4s (Supplementary Fig. S3). The obtained K_D_ values (Supplementary Table S1) are similar to those observed for the interaction between DinG and the parallel G4s studied herein derived from human genome.

It has occasionally been observed that, although one would expect a helicase to recognize the preferred substrate with high affinity, the substrate with the highest affinity is not the one that is unwound more efficiently. This is not surprising as helicases carry out a complex and dynamic reaction and need not only to bind to a substrate, but actively moving along it. Therefore, in a parallel effort we evaluated the ability of DinG to unwind the same panel of G4s, using a biochemical assay. To avoid the difficulties to separate the folded and unfolded unimolecular G4s in canonical helicase gel-based assays, we opted for the two-step fluorescence-based helicase assay developed by Mendoza et al.^35^. The assay was originally developed for the Pif1 helicase and was very easily adapted to DinG, that has the same unwinding polarity.

**Figure 2:**
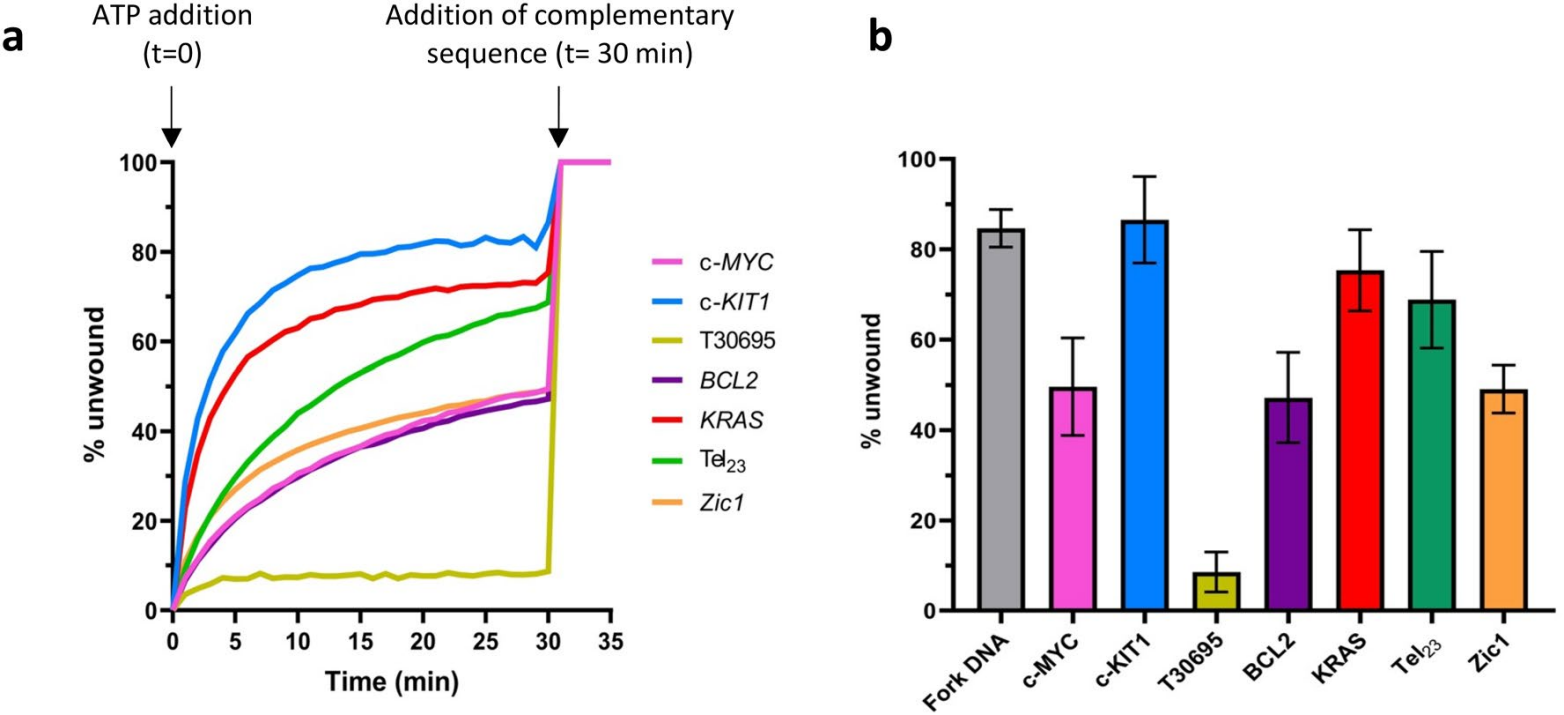
(**a**) Representative plots of fluorescence emission vs time for unwinding of selected DNA systems (S-c-*MYC*, S-c-*KIT1*, S-*T30695*, S-Tel_23_, S-*KRAS*, S-*BCL*2 and S-*Zic1*). ATP was added to begin the reaction (t = 0 min); the complementary strands (C-c-*MYC*, C-c-*KIT1*, C-*T30695*, C-Tel_23_, C-*KRAS*, C-*BCL2* and C-*Zic1* respectively) were added once the reactions reached a plateau (t = 30 min) as described^35^. (**b**) Quantification of the helicase activity of *E. coli* DinG against selected DNA systems; error bars indicate standard deviation of three independent experiments.

Experiments were performed using the same G4-forming DNA sequences analyzed by SPR. Using this assay (Fig. 2, Figs S4 and S5) we found that c-*KIT*1 G4 was almost completely unwound by DinG (around 85%) with the same efficiency shown for the DNA-fork (Fig. 2b). Our results also indicate that* KRAS* and Tel_23_ G4s were unwound with an efficiency of about 70–75%, while most of the other G4 structures (c-*MYC*, *BCL*2, EC6, EC7 and EC9) were unwound with an efficiency of about 50% (Fig. 2 and Fig. S5). Since our results differ from those reported in the literature showing that DinG was unable to unwind unimolecular G4^25^, we also tested the intramolecular G4 found in the *Zic*1 gene that was used for the previous study, and see some evidence for unwinding, although at a lower level than seen for the best G4 substrates (c-*KIT1* and *KRAS*). We think that the discrepancy can be explained by the relatively lower efficiency of DinG towards this particular substrate, and the different assay used. Indeed, the unwinding of unimolecular G4s can be more difficult to assess in gel-based assays due to a combination of effects (a strong tendency to refold, difficulties in separating the various species based on mobility, etc.).

A similar study carried out on Pif1 unwinding of different G4 structures showed some specificity, but to a much lower degree^35^, in line with the high efficacy of Pif1 as G4 helicase.

Consistently with the SPR data, the lowest unwinding activity was observed for the G4 structure of *T30695* (Fig. 2), which was shown to bind less efficiently; this is not surprisingly as (although we pointed out that we do not always find an exact correlation between binding affinity and unwinding efficiency) the first step of the helicase reaction mechanism is the recognition of the substrate, and when the affinity is too low, the helicase is unable to target the substrate and thus to unwind it. It had to be noted that *T30695* has a very different topological arrangement (a dimeric parallel structure), which is clearly distinct from all the other G4 substrates.

The unwinding activity of DinG towards the intramolecular G4 substrates (c-*KIT1, KRAS*, c-*MYC*, *BCL2*, *Zic1,* and Tel_23_) was further investigated in the presence of known G4 ligands, which are known to bind and stabilize G4 structures. Four well-characterized G4 ligands, Pyridostatin (PDS)^14^, PhenDC3^15^, BRACO-19^16^ and Netropsin^51^, were chosen to assess whether the helicase activity was affected. Among these, Netropsin was chosen for its capability to bind to the G4 grooves, to check if there are some differences in the DinG binding and/or in unwinding activity with respect to the other ligands.

**Figure 3:**
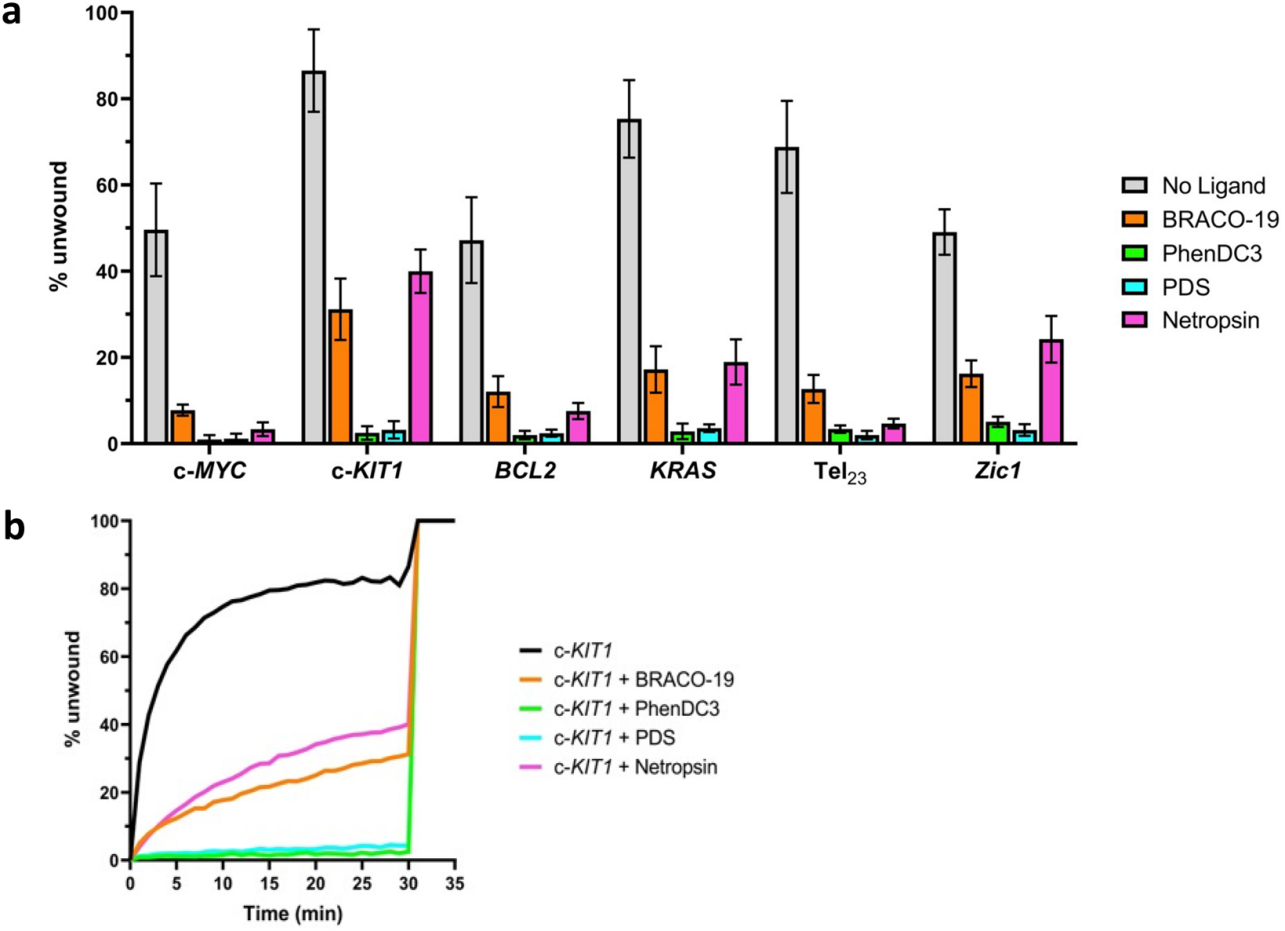
(**a**) *E. coli* DinG helicase activity against DNA systems S-c-*MYC*, S-c-*KIT1*, S-*BCL2*, S-*KRAS*, S-Tel_23_ and S-*Zic1* in the absence or presence of selected G4 ligands (BRACO-19, PhenDC3, PDS and Netropsin). Error bars indicate standard deviation of three independent experiments. (**b**) Example of typical real-time unfolding of S-c-*KIT1* system in absence or presence of selected ligands (BRACO-19, PhenDC3, PDS and Netropsin). ATP was added at t = 0; the complementary strand (C-c-*KIT1*) was added at t = 30 min, as described in^35^.

Not surprisingly, when the G4 structure was stabilized by the selected G4 ligands, in most cases the activity was abolished (Fig. 3 and Supplementary Fig. S6). Only in the presence of BRACO-19 and Netropsin some residual helicase activity is maintained, suggesting that these ligands have a lower impact on the helicase’s G4 unwinding. Conversely, PhenDC3 and PDS completely hinder the G4 unwinding by the helicase.

In a previous study, it was found that the DHX36 helicase unwinding activity towards c-*MYC* G4 was preserved in the presence of BRACO-19, NMM, and PhenDC3^36^. On the other hand, the unwinding activity of Pif1, another well-studied helicase, decreased in the presence of PDS, PhenDC3, and BRACO-19 bound to different G4s, with different ligands causing a different degree of inhibition, which was dependent on the specific G4 examined^35^. Similarly, whereas in the presence of PhenDC3 and PDS the DinG activity is abolished for all G4s, in the presence of BRACO-19 the effect is more pronounced for certain G4s than for others.

**Figure 4:**
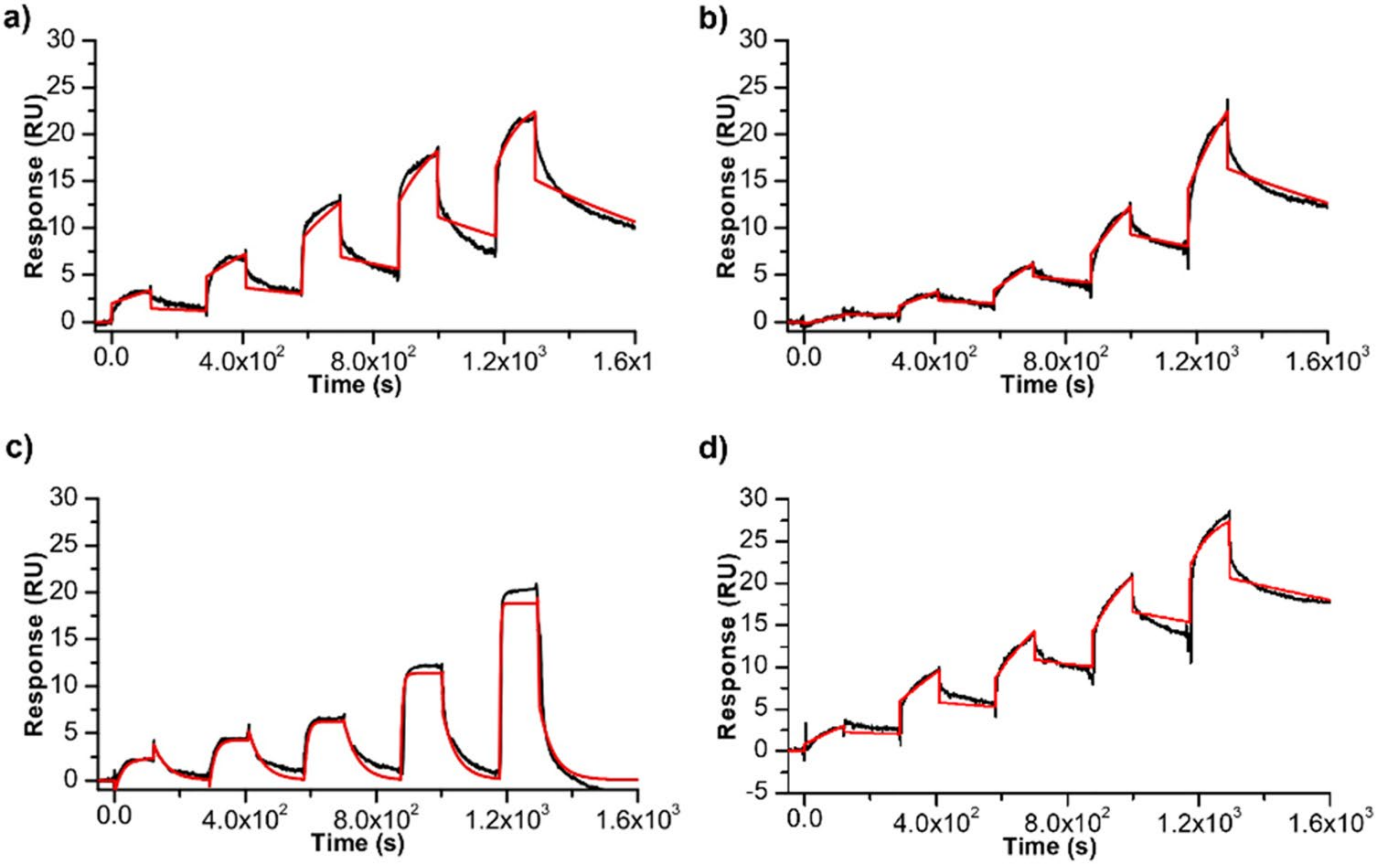
Time evolution SPR sensorgrams obtained at 25 °C by injection of increasing concentrations (from 0.062 to 1 µM) of c-*KIT*1 in the presence 1 mol equiv. of (**a**) PDS, (**b**) PhenDC3, (**c**) BRACO-19 and (**d**) Netropsin on the chip-immobilized DinG helicase.

To clarify whether the decreased activity of DinG is correlated to its lower capability to recognize and bind to the G4 when complexed to the ligands, SPR measurements were again carried out on the c-*KIT*1 G4, as the best substrate for unwinding. SCK experiments were performed on DinG-immobilized sensor chip flowing the c-*KIT*1 G4 in the presence of each of the four selected G4 ligands. Increasing concentrations of the [G4:ligand] (1:1) complex were flowed on the immobilized protein, as reported above. The sensorgrams are shown in Fig. 4 and relative data summarized in Table 3. An inspection of the data shows that the binding affinity of DinG for the c-*KIT*1 is affected but not completely hindered by the presence of BRACO-19, PhenDC3 and Pyridostatin, while the presence of Netropsin appears to have no significant impact on the G4 recognition by the helicase. This could be attributed to the different binding mode of Netropsin compared to the other investigated G4 ligands. To be noted, the change in binding affinity is not always closely related with the decrease in unwinding activity: while the lack of unwinding activity in the presence of PhenDC3 could be explained considering the decrease of DinG affinity for G4/ligand complex, the DinG binding to the G4 in the presence of BRACO-19 is comparable to that observed in the presence of PDS, but the effects on the unwinding activity are different in the two cases.

**Table 3:** SPR data for DinG interaction with c-*KIT*1 G4 in the absence and presence of ligands.

G4	k_on_ (M^–1^ s^–1^)^a^	k_off_ (s^–1^)^a^	K_D_ (nM)^b^
***c-KIT*1 G4**	1.2 × 10^4^	3.3 × 10^–4^	28.9
+ PDS	1.2 × 10^4^	1.1 × 10^–3^	98.3
+ PhenDC3	3.1 × 10^3^	8.3 × 10^–4^	266.1
+ BRACO-19	3.1 × 10^5^	0.023	73.9
+ Netropsin	1.4 × 10^4^	4.5 × 10^–4^	31.5

^a^Errors were within 5%.

^b^Errors were within 10%.

These findings could be explained by considering the different thermal stabilization properties induced by the ligands on the c-*KIT*1 G4 thermal stability, as indicated by results of CD melting experiments (Fig. S7). Indeed, a marked increase of the melting temperature of c-*KIT*1 G4 was observed already after the addition of 1 mol equiv. of PDS and PhenDC3 (Fig. S7a,b), while G4 stabilization by BRACO-19 is evident only in the presence of 2 mol equiv. of this ligand (Fig. S7c). Furthermore, DinG exhibits a comparable binding affinity for *c-KIT*1 G4 in the free and Netropsin-bound state; this result again correlates with CD melting data showing that Netropsin does not significantly affect the thermal stability of c-*KIT*1 (Fig. S7d). However, a partial inhibition of the unwinding is observed for c*-KIT*1 in the presence of Netropsin compared to c*-KIT*1 alone (Table 3).

## Conclusions

In conclusion, we were able to show for the first time that DinG binds with high affinity and resolves the biologically relevant unimolecular G4s, whereas previous reports suggested that only bimolecular G4s could be unwound^25,26^. Although the binding affinity of DinG for G4 does not drastically change from one G4 to another, the protein displays a helicase activity with different efficacy for the various G4s. The different behaviour of the helicase on c-*KIT1* compared to c-*MYC* G4, whose topological arrangement is very similar, suggests a correlation between the unwinding and the thermodynamic stability of the G4, with c-*MYC* G4 exhibiting a higher melting temperature than c-*KIT1*. The higher the stability, the lower the unwinding and vice versa^52–54^. At a more general level this result suggests that helicases display a selectivity that goes beyond the simple distinction between intramolecular and intermolecular G4s, or among the different G4 topologies; the possibility of a selective recognition/activity towards some but not other G4s should thus be taken into account when assessing the activity of a helicase towards G4s.

**Figure 5:**
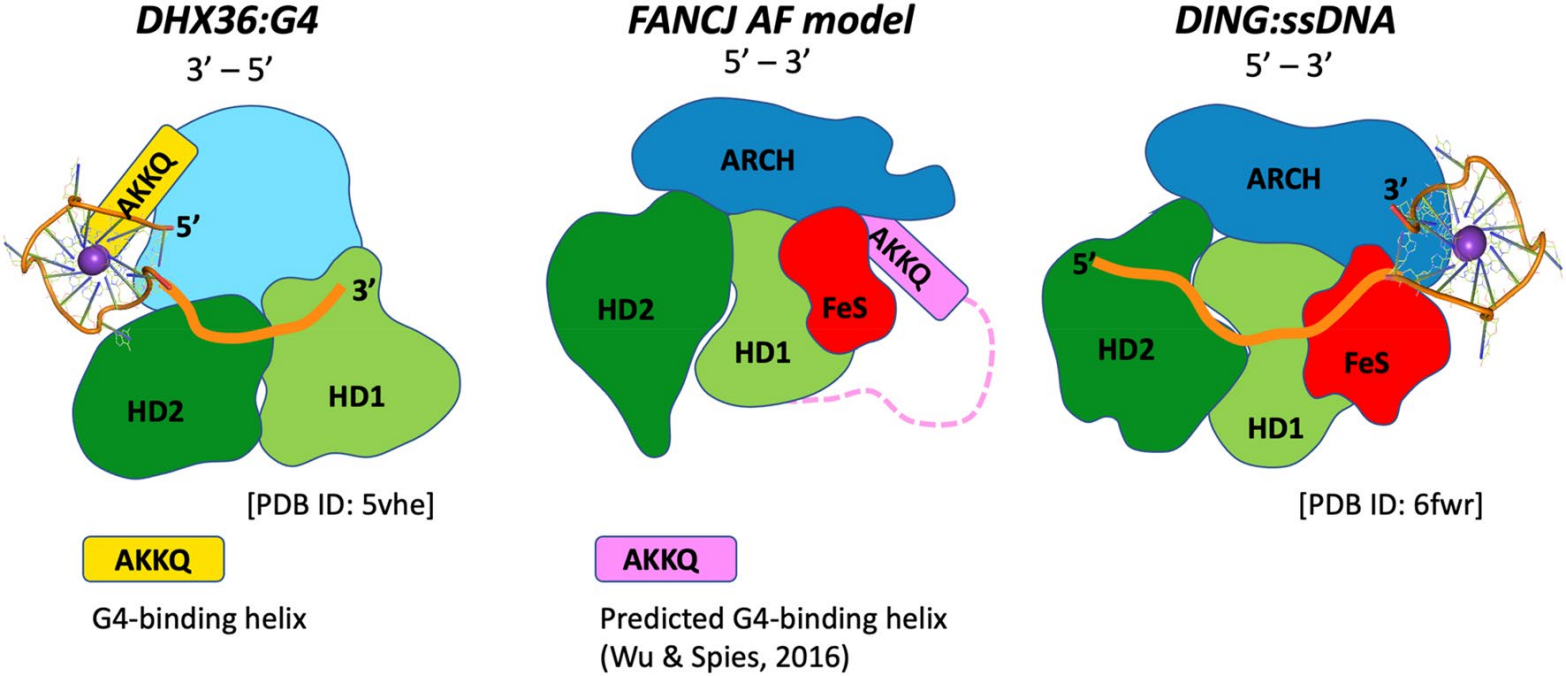
Schematic representation of the architecture of potential helicase:G4 complexes for enzymes belonging to the SF2 family. The left panel shows the crystal structure of the helicase DHX36 bound to a G4^32^, the central panel the AlphaFold predicted model^56^ for the human FancJ helicase, and the right panel the crystal structure of DinG bound to ssDNA^23^. The HD1 and HD2 domains forming the helicase catalytic core are shown in light and dark green respectively; in other colors, the domains that are peculiar to each family, and in orange the path of ssDNA. The DHX36 helix which plays a major role in G4 recognition (including the AKKQ sequence motif) is shown in yellow. It has been suggested that an equivalent helix is present in a FancJ-specific and partially unstructured insertion (shown in pink), that also contains a AKKQ motif, and was predicted to be involved in G4 recognition^55^; the AlphaFold model positions this helix between the FeS and the ARCH domains. This location is consistent with the crystal structure of the complex between DinG and ssDNA: a G4 has been placed on the [DinG:ssDNA] schematic diagram to illustrate the putative G4 binding region.

The fact that we don’t see a systematic correlation between binding and unwinding is consistent with the structural information available for the helicase:G4 complexes. Within Superfamily 2 helicase (to which DinG and the FeS helicases belong), the only three-dimensional model for a G4 bound to a helicase is that of the DHX36 helicase^32^. This structure shows that G4 recognition occurs in a different region of the protein from the groove engaging the single strand for the unwinding activity. The crystal structure of DinG bound to ssDNA^23^ shows the nucleic acid binding to a similar groove to DHX36, groove that could not accommodate anything more bulky, such a G4. A model had been proposed for FancJ:G4 recognition^55^: although the proposed binding region involves a helix located in an insertion in FancJ that is not present in DinG or other FeS helicases, AlphaFold models a possible position for this helix, suggesting that the G4 in FeS helicases could be located between the FeS domain and the ARCH domain. A schematic representation of the architecture of potential helicase:G4 complexes for proteins belonging to the SF2 family is shown in Fig. 5.

We would therefore expect DinG to similarly recognise G4s through a different region from the one engaged in unwinding, probably located adjacent to the FeS domain, providing a structural explanation for the differences we observe in binding and helicase activities, both in the absence and presence of ligands. Although the presence of G4 ligands does not necessary prevent the recognition of G4 by DinG, it can severely limit its unwinding activity; moreover, the effect is ligand-specific and G4-specific, with different ligands affecting the helicase activity by a different extent on various G4s. These results are important in view of designing new G4-ligands that can regulate unwinding activity.

Most of the work addressing the cellular function of DinG suggests a role in the resolution of R-loops^57^. However, this is very compatible with a parallel role in G4 resolution, as there is growing evidence of a widespread interplay between G4s and R-loops^52,58–60^ indicating that G4s are structurally compatible with R-loops and they can form simultaneously in vitro and in vivo, thus suggesting they act synergistically^59,61^.

As DinG is present in several pathogenic bacteria (including *Mycobacterium tuberculosis*, *Neisseria meningitidis* and *Neisseria gonorrhoeae*) a better understanding of the mechanism of action of the FeS helicase DinG can have a medical impact in terms of design of novel drugs. Moreover, as a model system for the more challenging eukaryotic FeS helicases, such as FancJ, DDX11 and RTEL1, which have all being implicated in G4 metabolism, the study of the interaction between DinG and G4s in the absence and presence of G4 ligands may provide a framework to better harness the potential of G4 stabilization in genome stability and cancer onset and development.

### Supplementary Information

Supplementary Information.

## Data Availability

The datasets used and/or analysed during the current study are available from the corresponding authors on reasonable request.
