# Supplementary material for: Exploring the G-quadruplex binding and unwinding activity of the bacterial FeS helicase DinG
Source: Sci Rep. 2023 Aug 3;13:12610. doi: 10.1038/s41598-023-39675-5 (PMC10400533; doi:10.1038/s41598-023-39675-5)
Supplement: Supplementary file 1 — Supplementary Information. [file 41598_2023_39675_MOESM1_ESM.pdf]

Supplementary data

Exploring the G-quadruplex binding and unwinding activity of the bacterial FeS helicase DinG

Elisa De Piante[1,2,‡], Federica D'Aria[2,‡], Luisa MR Napolitano[1], Jussara Amato[2], Simone Pirrello[1], Silvia Onesti[1,*], Concetta Giancola[2,*]

[1]Structural Biology Laboratory, Elettra-Sincrotrone Trieste S.C.p.A, 34149 Trieste, Italy
[2]Department of Pharmacy, University of Naples Federico II, 80131 Napoli, Italy

[‡]These authors equally contributed to this work.
*Authors to whom correspondence should be addressed: silvia.onesti@elettra.eu & giancola@unina.it

Table of contents

Table S1. Kinetics and thermodynamic parameters for the interaction of DinG helicase with G4s from *Escherichia coli*

Table S2. Substrates/systems sequence used for the two-step fluorescence-based helicase assay

Table S3. Oligonucleotides used to form the fork DNA, employed in the binding and helicase assays

Figure S1. CD spectra at 25 °C of G4s utilized in this study

Figure S2. CD spectra of G4s from *Escherichia coli* at 25 °C

Figure S3. Time evolution SPR sensorgrams for the interaction of DinG helicase with G4s from *Escherichia coli* at 25 °C

Figure S4. Typical fluorescence emission spectra

Figure S5. Quantitation of *E. coli* DinG helicase activity against selected DNA system from *Escherichia coli*. Typical fluorescence emission spectra

Figure S6. Typical real-time unfolding of G4s

Figure S7. CD melting experiments of c-*KIT1* with selected ligands

Table S1. Kinetics and thermodynamic parameters for the interaction of DinG helicase with G4s from *Escherichia coli*.

**Name**	Sequence	k_{on} ($M^{-1}s^{-1}$) [a]	k_{off} (s^{-1}) [a]	K_D (nM) [b]
**EC-6**	**5’-GGTGGGGAGGGGTAAGGGG-3’**	8.2 10^3	3.0 10^{-4}	36.7
**EC-7**	**5’- GGGGGAGGAGGACGGGGG -3’**	3.9 10^3	2.2 10^{-4}	56.9
**EC-9**	**5’- GGGGCGGGGTGGGTTGG -3’**	8.0 10^3	2.8 10^{-4}	34.9

[a] Errors were within 5%. [b] Errors were within 10%.

Table S2

Substrate/system sequences used for the two-step fluorescence-based helicase assay.

system[a]	Sequence
	5' – $(A)_{11}$-G4sequence-TATTCCGTTGAGCAGAG-3'- Dabcyl
Oligo FAM	3'-AAGGCAACTCGTCTC-5'-FAM
S-c-*MYC*	AGGGTGGGTAGGGTGGGT
S-c-*KIT*1	AGGGAGGGCGCGCTGGGAGGAGGG
S-T30695	GGGTGGGTGGGTGGGT
S-Tel_{23}	TAGGGTTAGGGTTAGGGTTAGGG
S-*KRAS*	AGGGCGGTGTGGGAATAGGGAA
S-*BCL2*	GGGCGCGGGAGGAATGGGCGGG
S-*Zic1*	GGTGGGGGGGCGGGGGAGGCCGGG
S-EC6	GGTGGGGAGGGGTAAGGGG
S-EC7	GGGGGAGGAGGACGGGGG
S-EC9	GGGGCGGGGTGGGTTGG
Trap	5'-TTCCGTTGAGCAGAG-3'
C-c-*MYC*	5'-CTCTGCTCAACGGAATACCCACCCTACCCACCC-$(T)_{11}$-3'
C-c-*KIT*1	5'-CTCTGCTCAACGGAATACCCTCCTCCCAGCGCCCTCCC-$(T)_{11}$-3'
C-T30695	5'-TCTTGCTCAACGGAATACCCACCCACCCACCC-$(T)_{11}$-3'
C-Tel_{23}	5'-CTCTGCTCAACGGAATACCCTAACCCTAACCCTAACCCTA-$(T)_{11}$-3'
C-*KRAS*	5'-CTCTGCTCAACGGAATACCCCTCTTCCCTCTTCCCACACCGCCC-$(T)_{11}$-3'
C-*BCL2*	5'-CTCTGCTCAACGGAATACCCGCCCATTCCTCCCGCGCCC-$(T)_{11}$-3'
C-*Zic1*	5'-CTCTGCTCAACGGAATACCCGGCCTCCCCCGCCCCCCCACC$(T)_{11}$-3'
C-EC6	5'-CTCTGCTCAACGGAATACCCCTTACCCCTCCCCACC-$(T)_{11}$- 3'
C-EC7	5'-CTCTGCTCAACGGAATACCCCCGTCCTCCTCCCCC$(T)_{11}$-3'
C-EC9	5'-CTCTGCTCAACGGAATACCAACCCACCCCGCCCC$(T)_{11}$-3'

[a] "S" oligonucleotides are the substrates for DinG unwinding; "C" indicates the complementary strands.

Table S3

G-scrambled sequence used for SPR experiments and oligonucleotides used to form the fork DNA, employed in the binding and helicase assays. For the helicase assay a 6-FAM (6-Carboxyfluorescein) label was attached at 5’-end of D1, and a BHQ1 label (Black Hole Quencher 1) was attached at 3’-end of D2.

Name	Sequence
G-scrambled	5’ GTGTGTGTGTGTGTGTGTGTGTGTGTGTGT 3’
D1	5’-CTACTACCCCCACCCTCACAACCTTTTTTTTTTTTTT-3’
D2	5’-TTTTTTTTTTTTTTGGTTGTGAGGGTGGGGGTAGTAG-3’
Cap1	5’-CTACTACCCCCACCCTCACAACC-3’

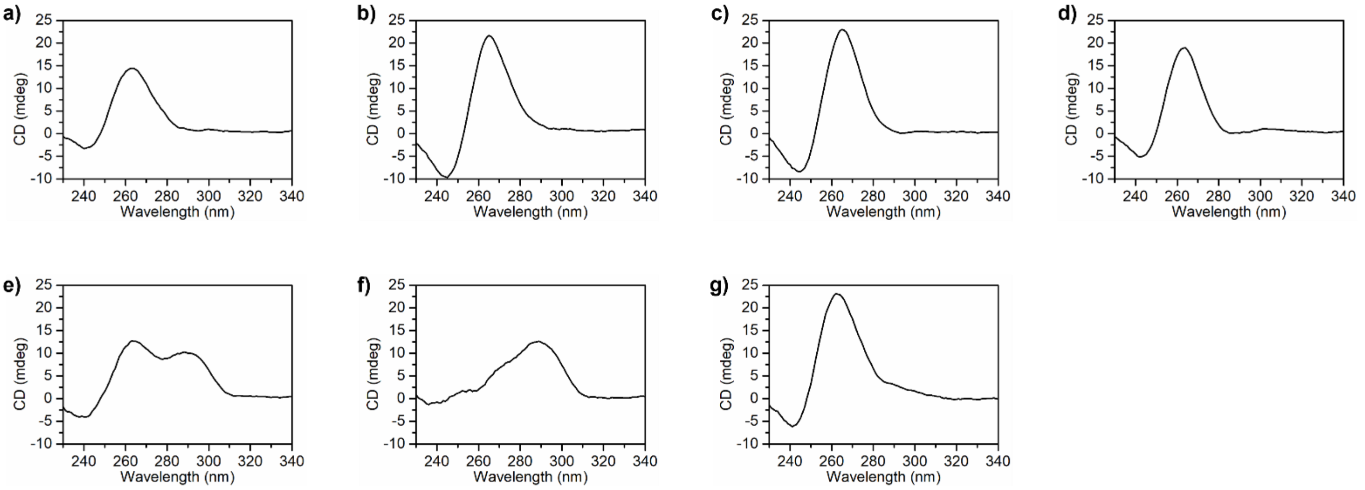

Figure S1. CD spectra of **a)** c-*KIT*1, **b)** c-*MYC*, **c)** *KRAS*, **d)** T30695, **e)** *BCL2*, **f)** Tel_{23}, **g)** *Zic1*

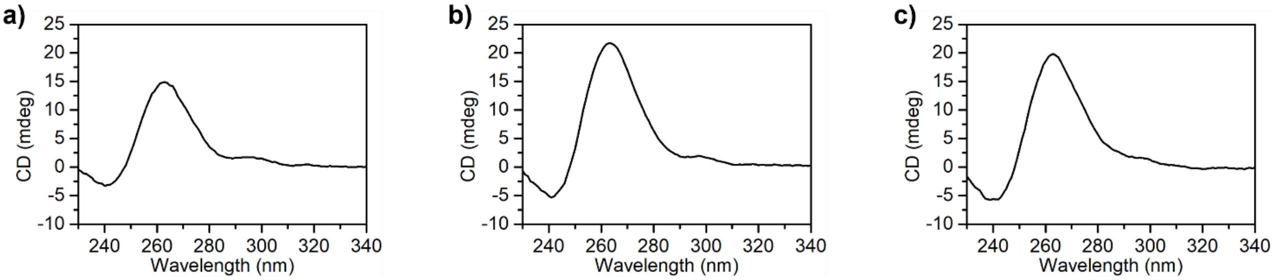

Figure S2. CD spectra of **a)** EC-6, **b)** EC-7, and **c)** EC-9

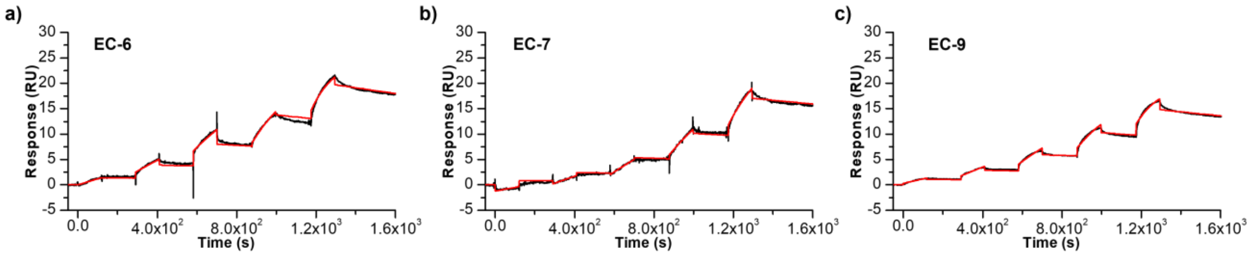

Figure S3. Time evolution SPR sensorgrams obtained at 25 °C by injection of increasing concentrations (from 0.062 to 1 μM) of three bacterial G-quadruplexes **a)** EC-6, **b)** EC-7, **c)** EC-9 on the chip-immobilized DinG helicase.

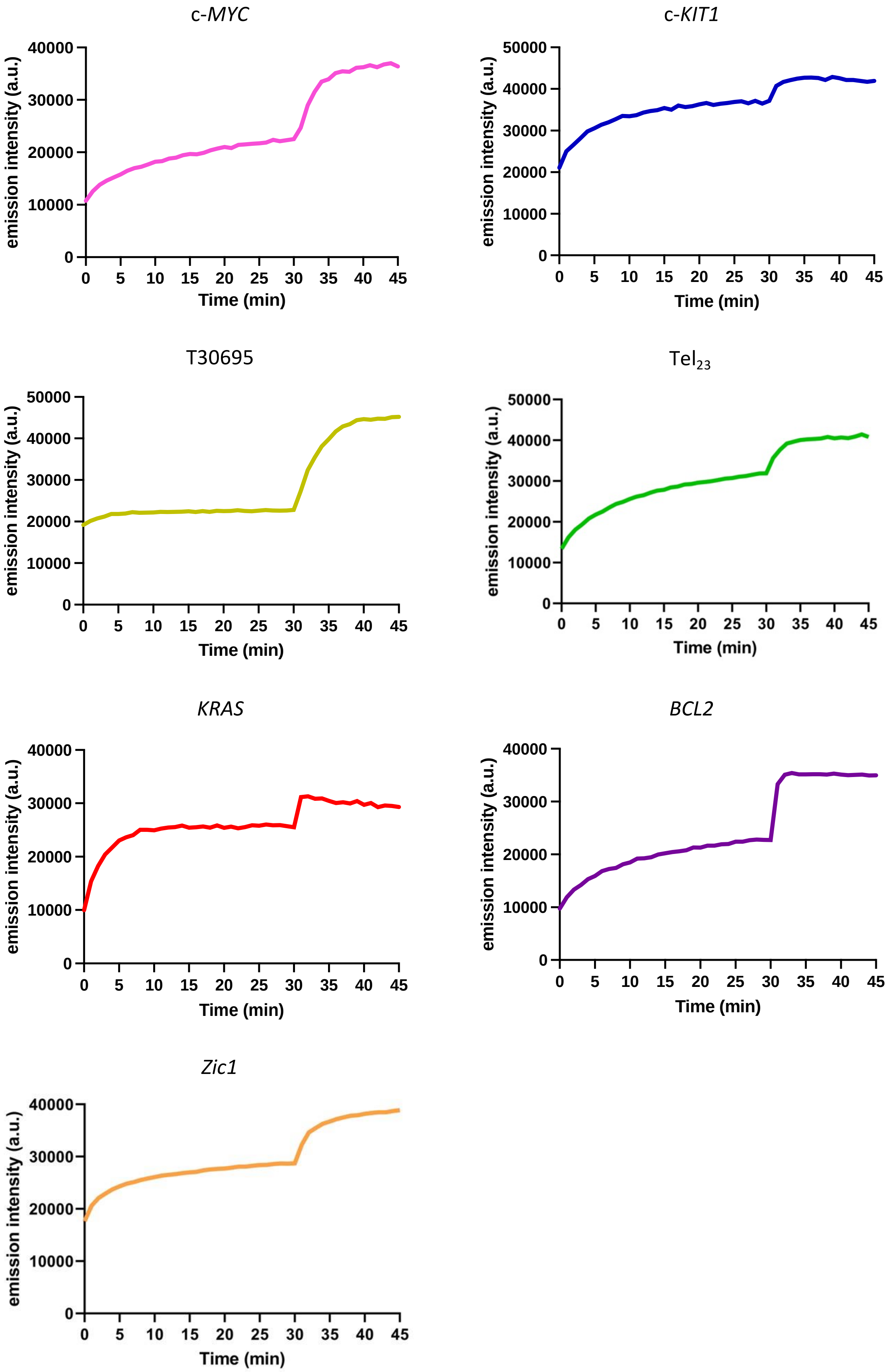
c-MYC
emission intensity (a.u.)
Time (min)
c-KIT1
emission intensity (a.u.)
Time (min)
T30695
emission intensity (a.u.)
Time (min)
Tel23
emission intensity (a.u.)
Time (min)
KRAS
emission intensity (a.u.)
Time (min)
BCL2
emission intensity (a.u.)
Time (min)
Zic1
emission intensity (a.u.)
Time (min)

Figure S4. Typical fluorescence emission spectra**.** Two-step unwinding process of selected DNA system (S-c-*MYC*, S-c-*KIT1*, S-*T30695*, S-Tel_{23}, S-*KRAS*, S-*BCL2* and S-*Zic1*) after (1) ATP addition (t= 0) and (2) addition of the relative complementary sequence (t= 30 min) (C-c-*MYC*, C-c-*KIT1*, C-*T30695*, C-Tel_{23}, C-*KRAS*, C-*BCL2* and C-*Zic1* respectively).

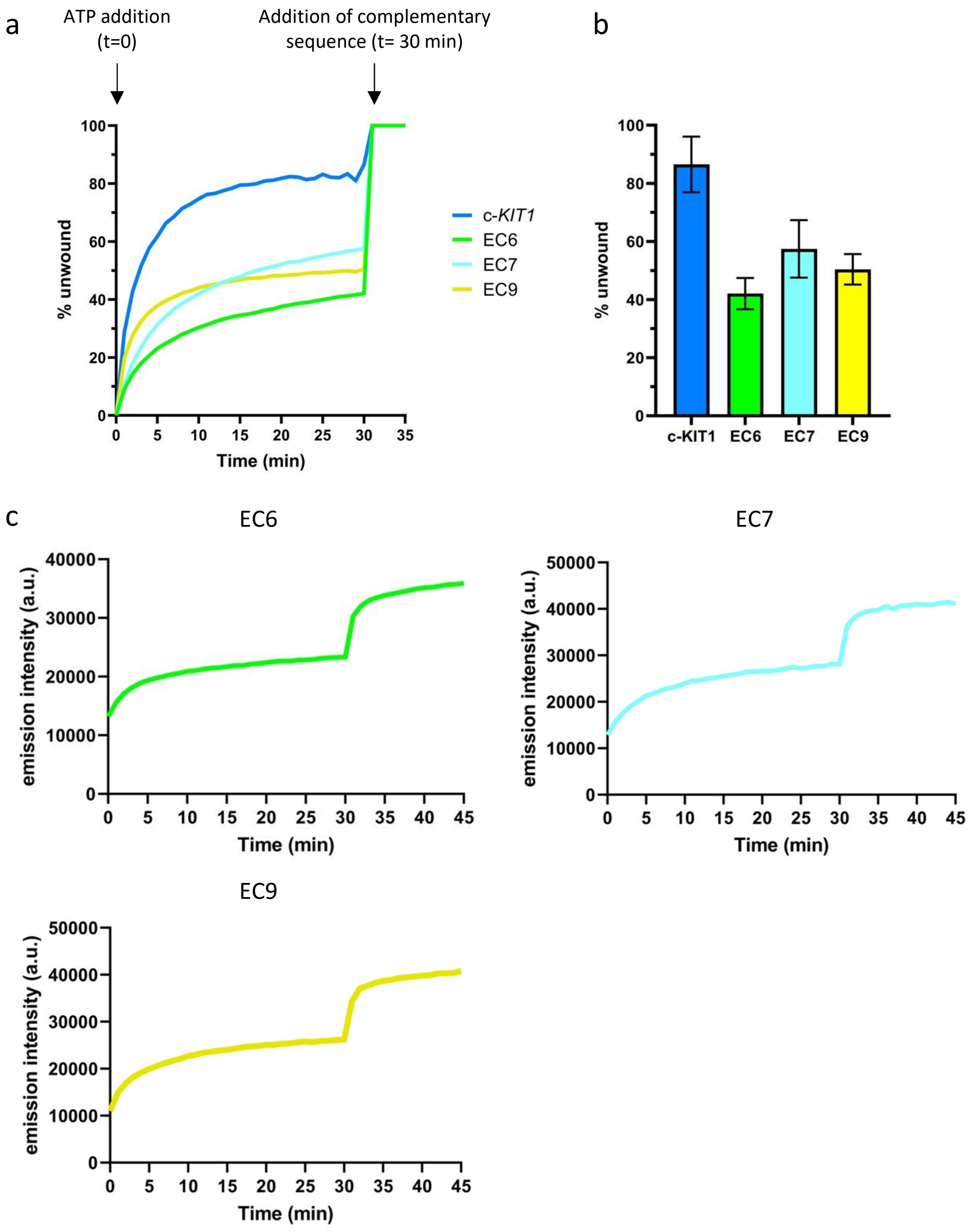

a
ATP addition
(t=0)
Addition of complementary
sequence (t= 30 min)
% unwound
Time (min)
c-KIT1
EC6
EC7
EC9
b
% unwound
c-KIT1
EC6
EC7
EC9
c
EC6
emission intensity (a.u.)
Time (min)
EC7
emission intensity (a.u.)
Time (min)
EC9
emission intensity (a.u.)
Time (min)

Figure S5. a) Representative plots of fluorescence emission vs time for unwinding of selected DNA systems (S-c-*KIT1*, S-EC6, S-EC7 and S-EC9). ATP was added to begin the reaction (t= 0 min); the complemetary strands (C-c-*KIT1*, C-EC6, C-EC7 and C-EC9 respectively) were added once the reactions reached a plateau (t= 30 min). **b)** Quantitation of *E. coli* DinG helicase activity against selected DNA systems; error bars indicate standard deviation of three independent experiments. **c)** Typical fluorescence emission spectra. Two-step unwinding process of selected DNA system (S-EC6, S-EC7 and S-EC9) after (1) ATP addition (t= 0) and (2) addition of the relative complementary sequence (t= 30 min) (C-EC6, C-EC7 and C-EC9 respectively).

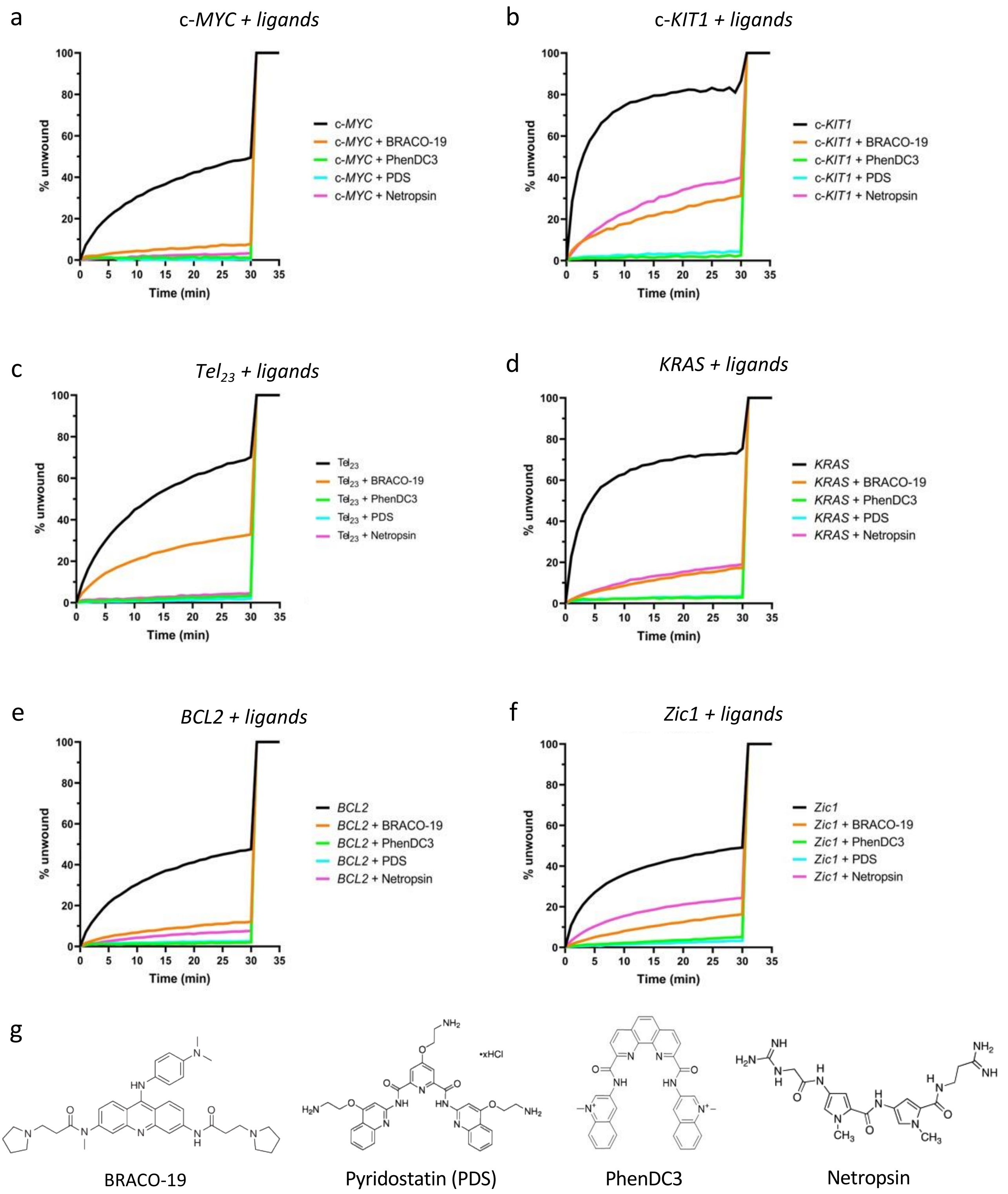

Figure S6. Typical real-time unfolding of **(a)** S-c-*MYC*, **(b)** S-c-*KIT1*, **(c)** S-Tel_{23}, **(d)** S-*KRAS*, **(e)** S-*BCL2*, **(f)** S-*Zic1* systems in absence or presence of selected ligands (BRACO-19, PDS, PhenDC3, Netropsin). ATP was added at t= 0; the complementary strand (C-c-*MYC*, C-c-*KIT1*, C-Tel_{23}, C-*KRAS,* C-*BCL2* and C-*Zic1* respectively) was added at t= 30 min. **(g)** Structure of selected ligands.

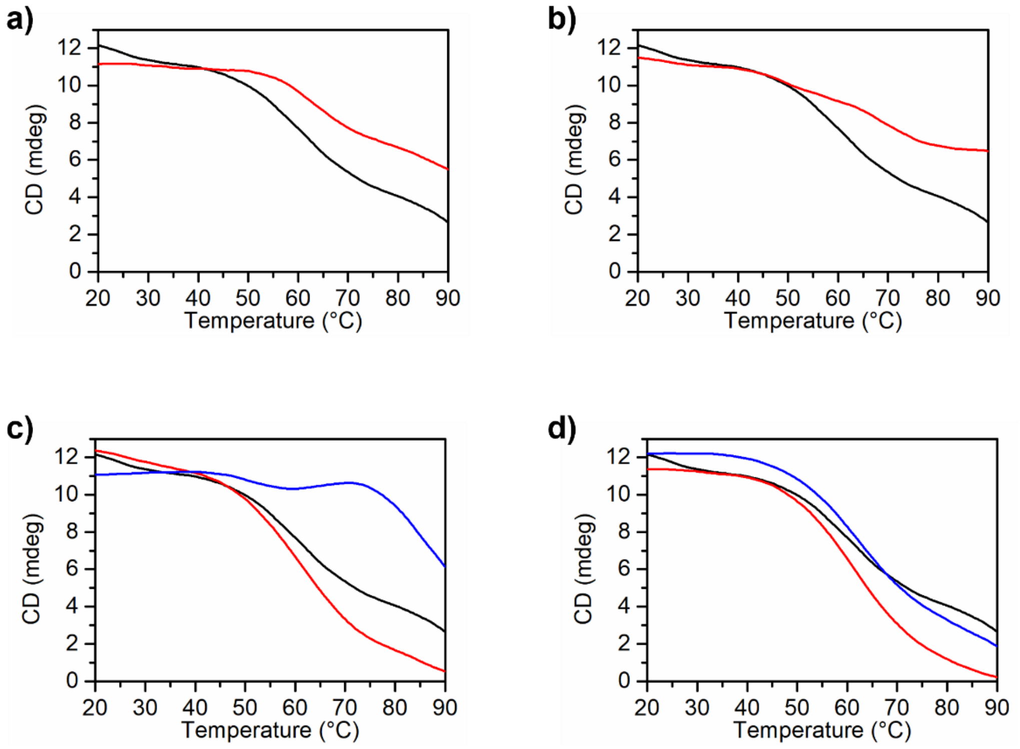

Figure S7. CD melting experiments of c-*KIT1* with 1 mol equiv. (red line) and 2 mol equiv. (blue line) of **a)** PDS, **b)** PhenDC3, **c)** BRACO-19 and **d)** Netropsin.
